# Hospital Length of Stay and Associated Factors in Adult Patients with Depression in Germany: A Cross-Sectional Study

**DOI:** 10.3390/jcm13154331

**Published:** 2024-07-25

**Authors:** Nimran Kaur, Marcel Konrad, André Hajek, Lee Smith, Karel Kostev

**Affiliations:** 1 Epidemiology, IQVIA, Bangalore 560 103, India; 2 Department of Health and Social, FOM University of Applied Sciences for Economics and Management, 60486 Frankfurt am Main, Germany; 3 Department of Health Economics and Health Services Research, University Medical Center Hamburg-Eppendorf, Hamburg Center for Health Economics, 20246 Hamburg, Germany; 4 Centre for Health, Performance and Wellbeing, Anglia Ruskin University, Cambridge CB1 1PT, UK; 5 Epidemiology, IQVIA, Unterschweinstiege 2-14, 60549 Frankfurt am Main, Germany; 6 University Clinic, Philipps-University, 35043 Marburg, Germany

**Keywords:** hospitalization, depression, hospital length of stay, depression, Germany, obesity, vitamin D deficiency, sleep disorders

## Abstract

**Objective:** The aim of the present study was to evaluate the hospital length of stay (LoS) and its associated factors among adult patients hospitalized with depression in Germany. **Methods*:*** This cross-sectional study included all adults (≥18 years) hospitalized with depression from January 2019 to December 2023 treated in 36 hospitals across Germany. The primary outcome was patients’ hospital LoS in days. The associations between age, sex, depression severity, co-diagnoses, hospital, and hospital LoS were analyzed using hierarchical multivariable linear regression models. **Results*:*** A total of 6579 patients (mean age 46.6 ± 17.7 years) with 8965 hospitalizations for depression were available. The mean hospital LoS was 35.2 days. Severe depression (+4.9 days) was associated with a longer hospital LoS, with moderate depression as the reference. Older age was positively associated with a longer hospital LoS. Vitamin D deficiency (+9 days), lipid metabolism disorders (+8 days), obesity (+8 days), sleep disorders (+7 days), and reaction to severe stress and adjustment disorders (+5 days) were also significantly associated with hospital LoS. **Conclusions*:*** In patients with depression, higher depression severity, advanced age, vitamin D deficiency, lipid metabolism disorders, obesity, sleep disorders, reactions to severe stress, and adjustment disorders were associated with a longer hospital LoS. Addressing these factors through comprehensive and integrated care strategies could help optimize hospitalization duration and improve overall patient outcomes.

## 1. Introduction

Depression is the most common mental health problem [1], frequently requiring hospital care [2]. Depression can be categorized with respect to its severity (from mild to severe) and duration (from months to years) [3]. Patients affected by depression may present with diverse signs and symptoms, including having a low mood, feeling worthless or guilty, impairment in concentration, absence of interest in daily activities, lack of energy, variations in appetite or weight, hypersomnia or insomnia, psychomotor delays or agitation, and thoughts of death or suicidal ideation and attempts [4]. A recent Italian study concluded that depression is intertwined with low-grade systemic inflammation that leads to frequent hospitalizations and higher mortality [5]. Individuals diagnosed with depression were found to be 2.4 times more prone to be hospitalized for complications (such as diabetes, bacterial pneumonia, chronic obstructive pulmonary disease, congestive heart failure, urinary tract infection, etc.), which improve when they receive timely and appropriate care [2]. A prospective cohort study including patients treated in multiple centers across Germany noted that nearly two in every ten hospitalized individuals were depressed [6].

The average hospital length of stay (LoS) increases for a combination of psychiatric disorders globally, particularly in Korea (8.8 ± 9.5 days) [7], Ethiopia (15 to 36 days) [8], Switzerland (33 days) [9], England (7 to 44.5 days) [10], and Germany (37 to 64 days) [11]. However, data are lacking about the associations between hospital LoS and distinct types of psychiatric disorders such as depression across different age groups, sexes, ethnicities, geographies, etc. [12]. A 14-year longitudinal study of Austrian patients aged ≥15 years concluded that the number of hospital admissions for patients with moderate depressive occurrences increased from 55 years of age, peaked at 65 years of age, and declined after that [12]. At the same time, the world’s aging population may lead to a saturation of available health services [6]. It has also been observed that depressive episodes are significantly more common among women than men [12]. Therefore, receptiveness to mental health therapies and utilization of health services may differ for various ages and sexes [13].

Depression is usually co-diagnosed with other chronic diseases [6]. Numerous studies explore the associations between depressive symptoms and disability, functional limitations, reduced quality of life, morbidity (cancer, cardiovascular diseases, diabetes, obesity, etc.), and even mortality [14]. The presence of depression in patients increased the LoS for those with Crohn’s disease (+8.2 days), ulcerative colitis (+6.1 days) [15], and heart failure (+3.3 days) [16], and also among those hospitalized for surgical procedures (+2.4 days) [17]. A meta-analysis suggested that depressive symptoms may result in hospitalization and are independently associated with an extended hospital LoS and a higher risk of readmission [1,18]. 

Thus, gaining a more in-depth understanding of the association between depressive symptoms and hospitalization is imperative because of the significant cost of continuous hospital LoS, especially for prolonged admissions [19]. As existing mental healthcare resources in Germany are not distributed based on the severity of the symptoms, patients with severe depression are often either insufficiently treated or kept waiting for psychotherapy for a prolonged duration [20], which may increase disease burden. There is a clear dearth of studies examining the association between hospitalization and patients’ emotional status and well-being [21]. In light of this, the aim of the present study is to evaluate hospital LoS and its associated factors among adult patients hospitalized with depression in Germany.

## 2. Methods

### 2.1. Study Design and Period

The present cross-sectional study was based on hospital database (IQVIA, Frankfurt am Main, Germany) data collected between January 2019 and December 2023. This database included data from 36 hospitals across Germany pertaining to some 1,223,696 hospitalizations. Participating hospitals were maximum care (i.e., large institutions with usually more than 1000 beds), primary care, specialized care, standard care, and university hospitals. 

### 2.2. Data Classification

The data collected by IQVIA include standardized information that the hospitals transfer to the Institute for the Hospital Remuneration System (InEK) under Section 21 of the German Hospital Fees Act (*Krankenhausentgeltgesetz* [KHEntgG]). The information for each hospitalization includes data on primary diagnoses, secondary diagnoses, and procedures performed during the hospitalization. Diagnoses were coded using the International Classification of Diseases, 10th revision (ICD-10), while hospitalization-related procedures were coded using the OPS classification (*Operationen- und Prozedurenschlüssel* in German). Finally, these routine data are sent to IQVIA regularly and in an anonymized format.

### 2.3. Study Population

The study included patients aged 18 or above hospitalized for depression (ICD-10: F32, F33) in Germany. All hospitalizations were included in cases where there were several hospitalizations for the same individual during the study period.

### 2.4. Study Outcome

The outcome of the study was the duration of hospital stay in days.

### 2.5. Study Variables

The study variables were age (in years; continuous and categorical variable [i.e., <18, 18–30, 31–40, 41–50, 51–60, >60]), sex (i.e., female and male), and depression severity (i.e., moderate depression [ICD-10: F32.1, F33.1] and severe depression [ICD-10: F32.2, F32.3, F33.2, F33.3]); co-diagnosis occurred and was documented in at least 5% of the study population (i.e., hypertension [ICD-10: I10], overweight and obesity [ICD-10: E66], thyroid gland disorders [ICD-10: E00–E07], lipid metabolism disorders [ICD-10: E78], diabetes mellitus [ICD-10: E10–E14], vitamin D deficiency [ICD-10: E55], alcohol-related disorders [ICD-10: F10], non-phobic anxiety disorders [ICD-10: F41], reaction to severe stress and adjustment disorders [ICD-10: F43], somatoform disorders [ICD-10: F45], sleep disorders [ICD-10: F51, G47], and specific personality disorders [ICD-10: F60]). The number of patients with mild depression [ICD-10: F32.0, F33.0] was small (0.3%) and thus not included in the analysis.

### 2.6. Statistical Analyses

The variables were described in the population using absolute numbers (percentages), except for age, which was represented as mean and standard deviation. In addition, the mean (standard deviation) hospital LoS (in days) was studied in the overall sample and for age, sex, and depression severity. Finally, associations between the variables (independent variables) and hospital LoS (dependent variable) were analyzed using hierarchical linear regression models adjusted for age, sex, depression severity, co-diagnoses, and hospital. The results of the linear regressions are displayed as ß-coefficients (differences in days) with *p*-values. All missing data were excluded from the analyses. Two-sided *p*-values lower than 0.05 were considered statistically significant. All analyses were performed using SAS version 9.4 (SAS Institute, Cary, NC, USA).

## 3. Results

This study included 6579 patients with 8965 hospitalizations for depression. More than 95% of the hospitalizations occurred in 12 of the 36 hospitals. The demographic characteristics of the study population are displayed in Table 1. Participants’ mean (standard deviation) age was 46.6 ± 17.7 years, and most patients (59.6%) were women. The majority (76.2%) of patients suffered from severe depression, while 23.2% suffered from moderate depression. Finally, the three most common co-diagnosed conditions were hypertension (27.8%), thyroid gland disorders (14.1%), and vitamin D deficiency (13.1%). 

**Figure 1:**
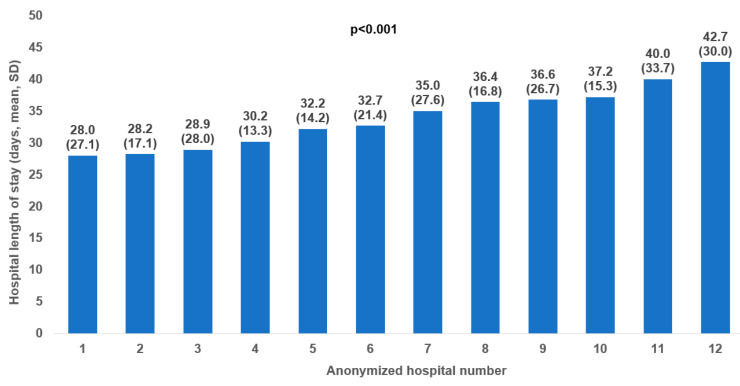
Hospital length of stay by hospital.

**Figure 2:**
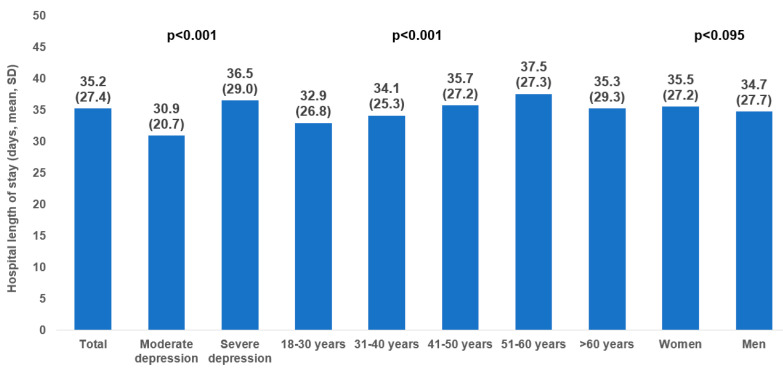
Hospital length of stay in the overall population and by age, sex, and depression severity.

The mean ± standard deviation LoS varied between 28 ± 27.1 days and 42.7 ± 30 days depending on the hospitals, as shown in Figure 1. The mean ± standard deviation hospital LoS was 35.2 ± 27.4 days in the whole sample, 30.9 ± 20.7 days for patients with moderate depression, and 36.5 ± 29 days for those with severe depression. Hospital LoS increased from 32.9 ± 26.8 days in people aged 18–30 years to 27.5 ± 27.3 days in those aged 51–60 years. In terms of sex, the figures were similar in women (37.5 ± 27.2 days) and men (34.7 ± 27.7 days)—Figure 2.

Hospitals had the strongest association with LoS from +6.5 to +11.6 days, depending on the hospital, as shown in Table 2. The results of the linear regression indicated that age was positively associated with hospital LoS, i.e., ß = +1.5 days in the 31–40 years age group, ß = +2.4 days in the 41–50 years age group, and ß = +3.3 days in the 51–60 years age group when the youngest age group (18–30 years) was taken as the reference. In terms of depression severity, severe depression (ß = +4.9 days) was associated with an extended hospital LoS compared to moderate depression. Furthermore, we identified seven co-diagnoses—specifically, vitamin D deficiency (ß = +9 days), lipid metabolism disorders (ß = +8.1 days), obesity (ß = +8.1 days), sleep disorders (ß = +7.2 days), and reactions to severe stress and adjustment disorders (ß = +4.7 days)—that were significantly and strongly associated with a longer hospital LoS. 

## 4. Discussion

The present cross-sectional study demonstrates that hospital LoS increased with depression severity and with growing age, i.e., from 18 years old to 60 years. Finally, vitamin D deficiency, lipid metabolism disorders, obesity, sleep disorders, reaction to severe stress, and adjustment disorders are strongly associated with prolonged hospital LoS.

An extended hospital LoS is strongly associated with the severity of depression among patients in previous studies [22], as also observed in our study. In addition, LoS can be prolonged in patients suffering from recurrent depressive disorders or impairment of social functioning and may also be impacted by the severity of their depression [11]. 

The mean hospital LoS in this study was similar to the findings of existing studies from Greece among patients with depression aged 18 to 65 years (42 days) [23] and patients of all ages from China (34 ± 7 days) [24]. In addition, the age-based analysis of hospital LoS in our study revealed that older adults had a prolonged hospital LoS, in contrast to the survey by Bakola et al. in 2024 [23], which ascertained that older age was inversely related to hospital LoS, and the study by Cheng et al. in 2022, who found no significant association between LoS and participant age [24]. However, a Romanian study noticed that older age was positively associated with longer hospital stays, similar to our observations [25]. The differences in these findings might be due to the inclusion criteria (all psychiatric disorders versus only people with diabetes versus major depressive disorders), study design (retrospective versus prospective), and geographical locations (Greece versus Romania versus China). Importantly, the presence or absence of comorbidities further influences the prognosis and therapeutic outcomes in hospitalized patients. 

AbuRuz et al., in 2021, estimated that the mean LoS for women (17.5 ± 12.7 days) is considerably longer than that for men (10.3 ± 9 days) through all depressive episodes [26]. However, the current study did not find any significant differences in hospital LoS between the two sexes, which was also the case in another retrospective survey from China focusing on hospitalized patients [24]. These differences might be due to the inclusion of patients with heart diseases only, the study design, and the geographical location (Jordan) in the former study. Sex-related differences among patients with depression may be due to inordinate hormonal instabilities during menopause and puberty, changes in the hypothalamic–pituitary–adrenal axis, and other psychosocial stressors among women [27].

The results of the present study resonate with those of another multi-site cluster randomized control trial from the United States of America that confirmed a strong link between depressive symptoms, hospitalization, and hospital-related outcomes, where most participants were ≥60 years old with numerous physical comorbidities [28]. A recent extensive cohort study including a population aged 63.3 ± 7.8 years from the United Kingdom and Finland determined that patients with severe or moderate depression suffered from bacterial infections, sleep disorders, mental disorders, behavioral disorders, neurological disorders, back pain, musculoskeletal diseases, osteoarthritis, ischemic heart disease, chronic obstructive bronchitis, endocrine and related internal organ diseases, diabetes, diseases of the blood and circulatory system, chronic kidney disease, chronic venous disorder, and foot ulcerations [25,29]. In the present study, patients with depression also had several co-diagnoses, though these patients were mostly <50 years old in contrast to both studies from the United States of America and the studies from the United Kingdom/Finland, which included patients aged 60 years or older [25,28,29]. 

In the present study, lipid metabolism disorders were associated with a higher LoS in patients with depression. These results align with those of a retrospective study from China showing a positive association between abnormal LDL values and hospital LoS [24]. A prospective study confirmed that the level of serum lipids among patients with major depressive disorders was associated with depression, its severity, and follow-up observations. Patients having major depressive episodes had increased values of triglycerides, low-density lipoprotein cholesterol, ratio of low-density lipoproteins/high-density lipoproteins, and total cholesterol [30]. Assuming that there is indeed a relationship between lipid metabolism disorders and depression severity, this may be an explanation for our findings showing a higher LoS in patients with depression and lipid metabolism disorders.

There is strong evidence linking vitamin D deficiency with several different diseases [31], and this draws attention to its impact on the relationship between hospital LoS and depression. Approximately 57.7% of the hospitalized patients (≥18 years) recruited in an Indian study were vitamin D-deficient [32]. The level of serum vitamin D is inversely correlated with clinical depression [33]. Evidence further suggests that vitamin D affects the central nervous system and acts as a mood modulator, affecting the brain receptors that are implicated in the pathophysiology of depression and anxiety disorders [34]. There are several reasons why hospitalized patients are vitamin D-deficient; this is mainly because of inadequate sun exposure as they spend more time indoors, are maintained completely under parenteral nutrition, have a vitamin D-deficient diet, have been diagnosed with specific health conditions (fat malabsorption disorders, cirrhosis, nephrotic syndrome, renal failure, gastric or small bowel resection), or are taking certain medications (rifampin, chronic corticosteroids, anticonvulsants) [35]. The median LoS for vitamin D-deficient patients increased by around 12 days (IQR: 8–15 days) [32], similar to our study (+9 days). By contrast, a Danish study found no significant link between vitamin D deficiency and hospital LoS [36]. Vitamin D deficiency is known to cause greater severity of chronic diseases, an increased risk of clinical instability [37], severe burden of morbidity, and low functional ability [38], consequentially leading to higher hospital mortality [39]. Thus, the association between hospital LoS and vitamin D in the present study is comparable to that in existing studies with participants suffering from specific medical conditions [38]. A meta-analysis of 308 studies from 2000 to 2022 found that Europeans were likely to be vitamin D-deficient when compared to other populations worldwide [31]. 

Furthermore, the sleep cycle is frequently disturbed in hospitalized patients. These disturbances could interfere with the patient’s recovery, prolong their hospital LoS, and cause dissatisfaction. The intrinsic factors acting as sleep interrupters are medical comorbidities, uncontrolled pain, physical inactivity, psychiatric conditions, and prescribed medications, while the external factors can be excessive exposure to artificial light, inappropriate humidity or temperature, a noisy environment, and medical interventions (night-time monitoring of vital signs, phlebotomy for diagnosis, and parental or oral medicine administration). Depriving the patient of good-quality sleep leads to improper memory consolidation, immune dysregulation, neuro-endocrine dysfunction, impaired cognition, and mood instability [40]. 

### Strengths and Limitations

The strengths of this study are a considerable sample size, a large selection of participating hospitals, and the enormous amount of data collected. As per the best of our knowledge, the present study is one of the first studies from Germany on this topic, and we have collected the latest and largest data. However, this study is subject to several limitations that need to be acknowledged. First, no data were available on the underlying reasons for hospitalizations to understand the delayed discharge of patients from the hospitals that lead to prolonged hospital LoS. Second, some of the patients might have been transferred to those hospitals that did not participate in this study, so this information was not documented. Further, this also means that hospital LoS may have been potentially underestimated. Third, data on socioeconomic status (e.g., patients’ education, socioeconomic status, income, etc.) and lifestyle-related risk factors (e.g., smoking habits, level of physical activity, alcohol consumption patterns or frequency, etc.) could not be included in our study. Fourth, we had no access to biological data; as a consequence, no information on the inflammatory markers of LoS could be evaluated. Fifth, the database contains only ICD-10 and operation and procedure (OPS) codes. These codes do not allow for differentiation between the first episode versus recurrent episodes of depression, which aid in evaluating the specifiers of depression (e.g., psychotic, melancholic, atypical, postpartum), type of treatment for depression (i.e., pharmacological, electroconvulsive therapy, psychotherapy, different combinations of these interventions), type of pharmacological treatment for comorbidities, and the severity of the comorbid disorders. Finally, as the present study is as a retrospective cross-sectional study, no causal effects of factors studied on the length of hospital stay can be concluded but rather only statistical associations.

## 5. Conclusions

In conclusion, patients with depression presenting with higher depression severity, advanced age, vitamin D deficiency, lipid metabolism disorders, obesity, sleep disorders, and reactions to severe stress and adjustment disorders tended to have a longer hospital LoS. Addressing these factors through comprehensive and integrated care strategies could help optimize hospitalization duration and improve overall patient outcomes.

## Figures and Tables

**Table 1:** Characteristics of the study population.

Variable	Hospitalizations(*n* = 8965)
*Age (in years)*	
Mean (standard deviation)	46.6 (17.7)
18–30	1883 (21.0)
31–40	1449 (16.2)
41–50	1575 (17.6)
51–60	2017 (22.5)
>60	2041 (22.8)
*Sex*	
Female	5339 (59.6)
Male	3626 (40,4)
*Depression severity*	
Moderate	2136 (23.8)
Severe	6839 (76.2)
*Co-diagnosis*	
Hypertension	2495 (27.8)
Obesity	470 (5.2)
Thyroid gland disorders	1262 (14.1)
Lipid metabolism disorders	661 (7.4)
Diabetes mellitus	754 (8.3)
Vitamin D deficiency	1177 (13.1)
Alcohol-related disorders	875 (9.8)
Non-phobic anxiety disorders	912 (10.2)
Reaction to severe stress and adjustment disorders	924 (10.3)
Somatoform disorders	741 (8.3)
Sleep disorders	788 (8.8)
Specific personality disorders	776 (8.7)

Data are absolute numbers (percentages) unless otherwise specified.

**Table 2:** Association between demographic and clinical variables and length of stay in patients hospitalized for depression (multivariable linear regression models).

Variable	ß-Coefficient (Difference in Days)	*p*-Value
18–30 years	Reference	
31–40 years	+1.5	0.101
41–50 years	+2.4	0.011
51–60 years	+3.3	<0.001
>60 years	+0.3	0.754
Female	Reference	
Male	−0.4	0.500
Moderate depression	Reference	
Severe depression	+4.9	<0.001
Hypertension	+2.5	<0.001
Obesity	+8.1	<0.001
Thyroid gland disorders	+1.5	0.065
Lipid metabolism disorders	+8.1	<0.001
Diabetes mellitus	−1.5	0.079
Vitamin D deficiency	+9.0	<0.001
Alcohol-related disorders	−1.0	0.321
Non-phobic anxiety disorders	−1.2	0.204
Reaction to severe stress and adjustment disorders	+4.7	<0.001
Somatoform disorders	+1.9	0.064
Sleep disorders	+7.2	<0.001
Specific personality disorders	+3.1	0.003

## Data Availability

The data and the code used for this study are available from the corresponding author upon request
